# Maternal, Perinatal and Neonatal Outcomes of Triplet Pregnancies According to Chorionicity: A Systematic Review of the Literature and Meta-Analysis

**DOI:** 10.3390/jcm11071871

**Published:** 2022-03-28

**Authors:** Mireia Bernal Claverol, María Ruiz Minaya, Irene Aracil Moreno, Santiago García Tizón, Pilar Pintado Recarte, Melchor Alvarez-Mon, Coral Bravo Arribas, Miguel A. Ortega, Juan A. De Leon-Luis

**Affiliations:** 1 Department of Public and Maternal and Child Health, School of Medicine, Complutense University of Madrid, 28040 Madrid, Spain; mruiz341060@salud.madrid.org (M.R.M.); irene.aracil@salud.madrid.org (I.A.M.); gineteca@gmail.com (S.G.T.); ppintado@salud.madrid.org (P.P.R.); jaleon@ucm.es (J.A.D.L.-L.); 2 Group of Pathophysiology in Women, Pregnancy, Labor and Puerperium, Health Research Institute Gregorio Marañón, 28040 Madrid, Spain; 3 Maternal and Infant Research Investigation Unit, Alonso Family Foundation (UDIMIFFA), 28009 Madrid, Spain; 4 Department of Medicine and Medical Specialties, Faculty of Medicine and Health Sciences, University of Alcalá, 28801 Alcalá de Henares, Spain; mademons@gmail.com (M.A.-M.); miguel.angel.ortega92@gmail.com (M.A.O.); 5 Ramón y Cajal Institute of Healthcare Research (IRYCIS), 28034 Madrid, Spain; 6 Immune System Diseases-Rheumatology and Oncology Service, University Hospital Príncipe de Asturias, CIBEREHD, 28801 Alcalá de Henares, Spain

**Keywords:** triplet pregnancies, chorionicity, trichorionic triamniotic (TCTA), neonatal complications, maternal outcomes, perinatal outcomes

## Abstract

Triplet pregnancies are rare events that affect approximately 93 in 100,000 deliveries in the world, especially due to the increased use of assisted reproductive techniques and older maternal age. Triplet pregnancies are associated with a higher risk of fetal and maternal morbidity and mortality compared to twins and singletons. Chorionicity has been proposed as a major determinant of perinatal and maternal outcomes in triplet pregnancies, although further evidence is needed to clarify the extent and real influence of this factor. Thus, the aim of this study was to conduct a systematic review of the literature and a meta-analysis of the maternal and perinatal outcomes of triplet pregnancies, evaluating how chorionicity may influence these results. A total of 46 studies with 43,653 triplet pregnancies and 128,145 live births were included. Among the main results of our study, we found a broad spectrum of fetal and maternal complications, especially in the group of monochorionic and dichorionic pregnancies. Risk of admission to NICU, respiratory distress, sepsis, necrotizing enterocolitis, perinatal and intrauterine mortality were all found to be higher in non-TCTA pregnancies than in TCTA pregnancies. To date, our meta-analysis includes the largest population sample and number of studies conducted in this field, evaluating a wide variety of outcome measures. The heterogeneity and retrospective design of the studies included in our research represent the main limitations of this review. More evidence is needed to fully assess outcome measures that could not be studied in this review due to scarcity of publications or insufficient sample size.

## 1. Introduction

Having triplets may be overwhelming news for many parents. It is indeed a diagnosis that is usually followed by multiple uncertainties and concerns that obstetric providers are expected to assess [1]. Given the limited data in the current published literature, physicians face a complex challenge when counselling patients on potential risks related to triplet pregnancies that will ultimately lead to potentially life-changing decisions in terms of management options. Clinical research in this field is thus necessary to identify the key aspects related to maternal and fetal morbidity and mortality in triplet pregnancies [2,3,4]

However, spontaneous triplet pregnancies are not frequent events. In fact, they occur spontaneously in 1/8000 pregnancies [5]. In recent decades, its incidence has not remained constant. With the upsurge in the use of assisted reproductive techniques (ART) in the 1980s and 1990s, multiple pregnancies increased exponentially, including triplets, by more than 400% during this period [6]. After a peak of 193.5 per 100,000 births was reached in 1998, its incidence began to decline significantly after recommendations to minimize multiple embryo transfers [7]. A 52% decrease was observed from 1998 to 2018, estimating a final incidence of 93 per 100,000 deliveries [8]. Currently, in the United States, it is estimated that 1 in 1880 pregnancies is a triplet pregnancy, and in 2020 there were 2738 triplet births [9]. In 2019, in Spain, there were 65 deliveries of triplet pregnancies [10]. 

A broad range of social and cultural determinants in the last decades have dramatically increased maternal age. Not only is advanced maternal age known to be associated with an increase in spontaneous multiple pregnancies, but it also leads to increased use of ART, which leads to higher rates of multiple pregnancies [6,11]. On the other hand, the slight decline of multiple pregnancies in recent years could be related to shifting strategies in ART protocols aimed at reducing the number of transferred embryos, as well as the standardization of fetal reduction procedures [12,13]. However, significant differences among countries should be expected, mainly due to cultural diversity regarding maternity and differences in access to ART and the protocols implemented locally.

Triplet pregnancies are associated with increased both fetal and maternal morbidity compared to singleton and twin pregnancies, including a higher risk of fetal growth restriction, preterm birth, and obstetric complications such as maternal diabetes or hypertensive disorders. Perinatal and neonatal death in triplets also appears to be more frequent [3,14,15,16,17].

Chorionicity has been suggested to be a key determinant of perinatal and maternal outcomes [14,16,18]. In fact, in twin pregnancies, monochorionicity has implications in terms of prematurity, fetal and neonatal morbidity, and mortality. This can be explained by the physiopathology underlying their shared circulation through vascular anastomoses and the possibility of developing a twin-to-twin transfusion syndrome (TTTS), selective restricted intrauterine growth, or fetal hemorrhage. Death or severe neurological morbidity of the survivor in cases of single intrauterine fetal death are also complications that can arise [19,20]. Some studies suggest that monochorionicity increases the risk of morbidity and mortality in triplet pregnancies [21], up to a two-fold increased risk when comparing monochorionic-triamniotic (MCTA) pregnancies to trichorionic-triamniotic (TCTA) pregnancies [14]. However, the impact of chorionicity on the prevalence of maternal, fetal, and neonatal morbidity and mortality has not been fully ascertained.

The objective of this study was to carry out a systematic review of the literature and a meta-analysis of the maternal and perinatal outcomes of triplet pregnancies, considering the factors that may influence these results and taking chorionicity into account.

## 2. Materials and Methods

The systematic review was carried out according to an a priori designed protocol and following the PRISMA “Preferred Reporting Items for Systematic reviews and Meta-Analysis” [22] and MOOSE “Meta-analyses and systematic reviews of Observational Studies (MOOSE) guidelines” guidelines [23]. This study was registered in the PROSPERO database [registration number: CRD 42020170836].

### 2.1. Information Sources and Search Strategy

The search was carried out from two electronic databases, PubMed and Embase, in January 2020, using combinations of the relevant MeSH terms, keywords, and variants of “triplet pregnancy”, “perinatal, maternal and neonatal outcome”. A database reference manager (EndNote) was used to incorporate all references.

### 2.2. Eligibility Criteria

To be considered eligible for inclusion, studies had to be written in English and published within the last 15 years (January 2005 to December 2020). Exclusion criteria were as follows: studies using animal models, studies not including abstracts, posters without a full article, studies not differentiating triplet pregnancies from other non-triplet multiple pregnancies, clinical trials or interventional studies regarding fetal reduction or laser therapy and their results as their main objective, systematic reviews, meta-analyses, case series, expert opinions or consensus, secondary analyses, guidelines, studies that excluded TCTA pregnancies, studies that only focused on a specific outcome, and studies only including patients with severe morbidity such as patients who were admitted to the NICU (to avoid a potential bias on the severity of the results).

### 2.3. Selection of Studies

All articles were analyzed independently by two authors (MBC and IAM), and if the title and abstract did not provide useful information for our review or were considered irrelevant for our main purpose, the article was discarded from our analysis. All articles were evaluated according to the inclusion and exclusion criteria previously mentioned. Inconsistencies were assessed by a third author (MRM), discussed by the reviewers, and a consensus was reached between the three investigators.

### 2.4. Data Collection

Data were collected using a standardized format. The data extracted from each article were the authors, year of publication, patient recruitment years, design and study type (single or multicentre, population or observational retrospective study), the country in which the study was conducted, the total number of pregnancies and live births included, the use of ART, and chorionicity, which divided pregnancies into TCTA and both dichorionic and monochorionic, encompassing the latter in the non-TCTA category.

Data regarding maternal outcome included maternal age, parity, body mass index, as well as maternal obstetric morbidities such as diabetes, hypertensive disorders (including gestational hypertension, preeclampsia, and eclampsia), restricted intrauterine growth or small for gestational age, twin to twin transfusion syndrome, congenital malformations, preterm labor, premature rupture of membranes, cervical cerclage, use of corticosteroids, antenatal bleeding and postpartum hemorrhage.

Data on perinatal outcome included mode of delivery (vaginal vs cesarean section), gestational age at the time of delivery, considering preterm birth (<37 weeks), very preterm birth (<32 weeks), and extremely preterm birth (<28 weeks); birth weight, considering low birth weight (<1500 g) and very low birth weight (<1000 g); APGAR at 1 min and 5 min; and sex.

The following data on neonatal outcome was also included: neonatal intensive care unit (NICU) admission and complications, such as respiratory distress syndrome (RDS), hyaline membrane disease, need for mechanical ventilation, use of surfactant, bronchopulmonary dysplasia (BPD), neonatal enterocolitis (NEC), neonatal sepsis, severe intraventricular hemorrhage (IVH type III and IV), retinopathy of prematurity (ROP) and composite neonatal morbidity.

Likewise, we explored mortality outcomes, such as miscarriage (under 22 weeks), intrauterine fetal mortality (IUFM; over 22 weeks), neonatal death (during the first 28 days after birth), and perinatal death, defined as the sum of both intrauterine and neonatal mortality.

First, a descriptive analysis of all the articles included in the systematic review was carried out. In addition, we analyzed which of the articles compared perinatal and maternal outcomes based on chorionicity, and included them in a meta-analysis for each of the available outcome measures. Finally, graphic representations of the results were obtained.

### 2.5. Risk of Bias Assessment and Statistical Analysis

Quality assessment of the included studies was performed using the Newcastle–Ottawa Scale (NOS) for case–control studies. According to this scale, each study is evaluated from three perspectives: the selection of the study groups (the representativeness of the exposed and unexposed cohort, their exposure, and the fact that the variable of interest was not present from the start of the study), the comparability of the groups, and the precision in the measurement of the variable of interest. According to NOS, a study can be awarded a maximum of one star for each item in the selection and outcome categories of interest and a maximum of two stars for comparability.

Different meta-analyses of proportions have been performed to estimate the combined incidence of our main variables of interest (maternal, perinatal, neonatal, and mortality outcomes) in TCTA and non-TCTA triplet pregnancies. Meta-analyses were only performed when at least 3 studies could be included. The heterogeneity of the studies was evaluated using the I^2^ statistic, considering a value >50% as indicative of high heterogeneity. Given the high variability found in many of the different studies, random effects models were chosen to estimate the pooled odds ratios. Under the assumption of random effects, in the weighting of the studies, both their own variability (intrastudy variability) and the variability among studies (interstudy variability) are considered. When random effects models are assumed, results are usually more conservative than those obtained under the assumption of fixed effects, obtaining wider confidence intervals for the combined odds ratios.

Potential publication bias was assessed using Egger’s regression asymmetry test for small study effects. Given the low number of studies included for each of the meta-analyses, the application of other formal tests for the evaluation of publication bias, such as the funnel plot, was not considered convenient.

All statistical analyses were performed using Stata 15.1 (StataCorp. 2017. Stata Statistical Software: Release 15. College Station, TX, USA: StataCorp LLC).

## 3. Results

**Figure 1:**
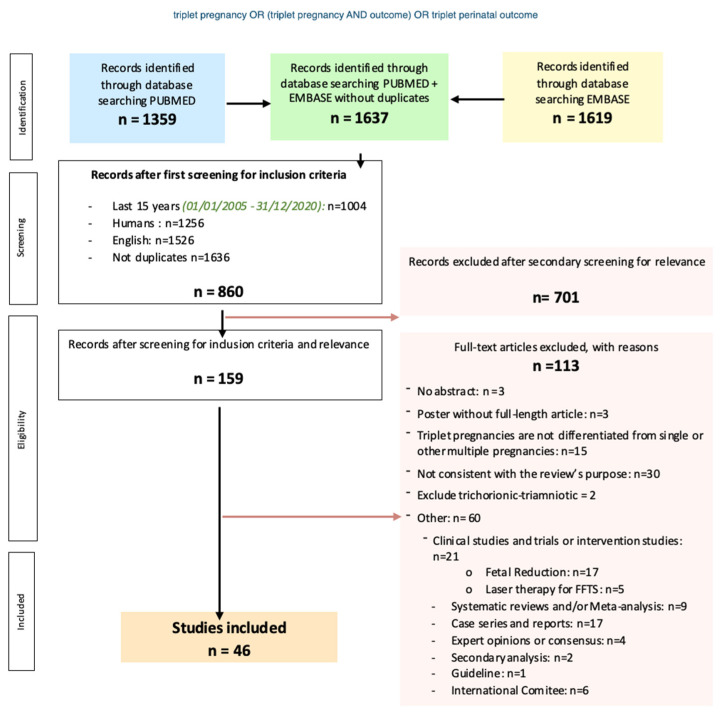
Flowchart of the systematic review, search strategy, inclusion and exclusion criteria.

The initial search revealed 1637 citations potentially eligible for inclusion, of which 860 met our inclusion criteria, and 701 were excluded based on their title or abstract. A total of 159 full manuscripts were reviewed, and 46 were ultimately included in the systematic review. Among the 46 studies, 12 were included in the meta-analysis, which studied the differences in maternal, perinatal, and neonatal outcomes when comparing TCTA with non-TCTA pregnancies. (Figure 1).

### 3.1. Descriptive Statistical Analysis

Table 1 shows the characteristics of the studies included in the systematic review. Most of them were published in North America (37%) and Europe (28.33%) and carried out in a single centre (54.4%). The predominant study design was retrospective observational (36; 78.3%), followed by 6 (13%) population studies and only 4 (8.7%) prospective observational studies. Sixteen (34.8%) of the studies were published from 2005 to 2009, 11 (23.9%) from 2010 to 2014, and 19 (41.3%) in the last five years.

Table 2, Table 3, Table 4, Table 5 and Table 6 show the data collected from the studies included in the systematic review.

A total of 46 studies with 43,653 triplet pregnancies and 128,145 live births were included. The number of outcome measures collected in the systematic review were: 6 gestational and maternal characteristics, 11 obstetric complications, 13 perinatal outcomes, 11 neonatal outcomes, and 6 mortality outcomes.

### 3.2. Maternal and Gestational Characteristics

Regarding the method of conception, 2962 (68.06%) pregnancies were obtained by ART. According to chorionicity, 64.1% were TCTA pregnancies, 24.88% were dichorionic triamniotic pregnancies, and 4.25% were MCTA pregnancies. It should be noted that three of the studies included in the review excluded monochorionic pregnancies [24,25,26]. The mean maternal age was 31.6 years (Table 2).

### 3.3. Obstetric and Maternal Outcomes

Table 3 describes the obstetric complications observed in the studies included in the systematic review. Preterm labor occurred in 26.39% of the pregnancies, and premature rupture of membranes in 11.52%. A total of 41.43% of the patients underwent cervical cerclage, although the heterogeneity was significant, varying from 3 to 63% in the 11 articles that included this variable. There were 4884.3 (34.92%) cases of intrauterine growth restriction or small for gestational age. TTTS complicated 2.79% of all the pregnancies included in the review. Two of the articles from the review specifically excluded pregnancies complicated by TTTS; thus, this percentage could be slightly underestimated [27,28]. Fetal malformations were diagnosed in 6.54% of pregnancies, although this was also an exclusion criterion for six of the included studies [25,28,29,30,31,32].

### 3.4. Perinatal and Neonatal Outcomes

There was an 88.98% rate of cesarean section. The mean gestational age at delivery was 32.3 weeks. In fact, 22134.7 (92.43%) deliveries were premature (<37 weeks), and 1475.7 (12.92%) were extremely premature (<28 weeks) (Table 4). A total of 4177 newborns (78.69%) were admitted to the NICU, with hyaline membrane disease (43.98%) and respiratory distress (25.51%) being the most common complications (Table 5).

### 3.5. Mortality

Miscarriages (<22 weeks) and intrauterine fetal mortality (IUFM) were close to 5%. On the other hand, the number of neonatal deaths was 44,089 (4.42% of live newborns for whom this variable was reported, which was 34.41% of live newborns). Four studies reported maternal mortality, which was one maternal death out of 721 pregnant women (0.14%) (Table 6).

### 3.6. Meta-Analysis Based on Chorionicity

A total of 13 articles were included in the meta-analysis, with 2188 pregnant women, 5790 fetuses, and 5441 live newborns. Data were collected on seven maternal and perinatal characteristics, eight neonatal, and three mortality outcome measures. Among all the variables studied, those that accounted for the largest number of studies and incorporated most data were perinatal mortality (ten studies with 5583 fetuses), followed by intrauterine mortality (nine studies with 5367 fetuses) and ART (nine studies with 1807 pregnant women). The variable for which the least data are available is very preterm birth (<32 weeks) (three articles with 225 pregnancies).

Table 7 describes the analysis of the maternal and perinatal characteristics. Chorionicity had no statistically significant differential impact on the mode of delivery or on prematurity. In the very preterm (<32 weeks) and very low birth weight infants, a tendency to lower risk was observed in TCTA pregnancies compared to non-TCTA pregnancies. Additionally, TCTA pregnancies were three times more likely to come from ART.

Regarding neonatal outcomes (Table 8), it is interesting to note that newborns from TCTA pregnancies had a significantly lower risk of being admitted to the NICU (OR 0.57; 95% CI 0.44–0.72) as well as of developing respiratory distress (OR 0.46; 95% CI 0.22). −0.97), sepsis (OR 0.57; 95% CI 0.37–0.89) and necrotizing enterocolitis (OR 0.32; 95% CI 0.15–0.69). However, no statistically significant differences were observed for very low birth weight (<1500 g), bronchopulmonary dysplasia, retinopathy of prematurity, or severe intraventricular hemorrhage stages III–IV.

Finally, in terms of mortality (Table 9), pregnancies without any monochorionic component (TCTA) showed a lower risk of both intrauterine (OR 0.29; 95% CI 0.14–0.62) and perinatal mortality (OR 0.32; 95% CI 0.20). −0.53).

Figure 2, Figure 3, Figure 4, Figure 5, Figure 6, Figure 7, Figure 8, Figure 9, Figure 10, Figure 11, Figure 12, Figure 13, Figure 14, Figure 15 and Figure 16 represent the forest plots of the characteristics and outcome measures previously described in Table 7, Table 8 and Table 9. As shown in these figures, due to the scarcity of articles that reported data on intrauterine growth restriction, APGAR < 7 at 5 min, and use of surfactant, the meta-analysis for these outcomes was not carried out.

## 4. Discussion

### 4.1. Main Findings

Both our systematic review and meta-analysis collected data on a wide range of outcome measures from a large population sample. This systematic review brings together a large population sample from a large number of studies (46 studies with a total of 43,653 triplet pregnancies and 128,145 live births), collecting information on 47 variables [14,16,21,24,25,26,27,28,29,30,31,32,33,34,35,36,37,38,39,40,41,42,43,44,45,46,47,48,49,50,51,52,53,54,55,56,57,58,59,60,61,62,63,64,65,66]. Similarly, this meta-analysis included many articles with a large total population sample (12 studies, 2188 pregnant women, 5790 fetuses, and 5441 live newborns), collecting up to 18 variables [14,16,21,24,26,33,36,41,44,56,57,63]. The vast majority of the studies have been published in the last 5 years (41.3%), which highlights the growing interest in triple pregnancies and their maternal and perinatal outcomes.

To date, the only meta-analysis published on perinatal morbidity and mortality in triplet pregnancies is the review conducted by Curado et al. [18], which concluded that chorionicity is a determining factor of perinatal morbidity in triplet pregnancies. Among its main findings, it is worth noting that non-TCTA triplet pregnancies show increased neonatal morbidity and higher intrauterine and perinatal mortality than TCTA pregnancies. Our main results are consistent with these findings and contribute to these statements, since the sample and the number of studies on which they are based have increased, in addition to enriching the existing literature with a greater number of variables.

Regarding our main results from the systematic review, it is striking that many articles lack information about baseline maternal characteristics, such as complications directly related to multiple pregnancies. Miscarriages in the first trimester are also underreported in comparison to the rest of the mortality analysis. Finally, the scarcity of data regarding maternal morbidity and mortality is surprising.

Regarding our meta-analysis, the main results are that the risk of admission to NICU or presenting neonatal complications such as respiratory distress, sepsis, or necrotizing enterocolitis is greater in non-TCTA pregnancies than in TCTA pregnancies. Perinatal and intrauterine mortality are also more frequent in non-TCTA triplet pregnancies.

### 4.2. General Considerations

#### 4.2.1. Comparison with Previous Literature

In the meta-analysis by Curado et al. [18], a smaller number of studies were analyzed with a smaller population sample (9 studies, 1373 pregnancies, 4119 fetuses, and 3669 live births) and a smaller number of variables. Curado et al. analyzed intrauterine, perinatal, and neonatal mortality and gestational age at birth as main outcomes. The secondary outcomes were composite neurological, respiratory and infectious morbidity. However, they did not carry out an extensive systematic review of the literature on maternal and obstetric characteristics or perinatal and neonatal complications separately.

#### 4.2.2. Socioeconomic and Geographic Determinants

The socioeconomic background has an undeniable role as a key determinant of maternal health and obstetric outcomes [67,68]. It has an impact on patients’ access to health resources, ART, tertiary care centres, and NICU availability. Ref. [69], In our study, most articles included had been published in Western countries (35 of the 46 publications of the systematic review), with only one study in Latin America, and no representation from the African continent. Furthermore, only one study was from an upper-middle-income country, and two were from a lower-middle-income country. This is even more apparent among the studies that were finally included in the meta-analysis, thus influencing the results of our study. It is therefore vital for physicians to counsel their patients according to their social, cultural, and economic background, taking into account the importance of such differences on morbimortality outcomes in triplet pregnancies. Moreover, considering that 68% of all triplet pregnancies in our systematic review were a result of ART, special attention must be paid to the selection of patients and strategies implemented to minimize the number of triplet pregnancies and their complications [70,71,72,73,74,75,76,77,78,79]. When analyzing the impact of ART on chorionicity in the meta-analysis, TCTA pregnancies were found to be up to three times more frequent, which aligns with the results of Fennessy et al., who found TCTA pregnancies to be four times more likely after ART [44].

#### 4.2.3. Maternal and Perinatal Characteristics

In the systematic review, the pooled proportion of the mode of delivery was 88% for cesarean section. Prematurity (before 37 weeks) occurred in 92.34% of all triplet pregnancies, with 40% being highly preterm (under 32 weeks). This is directly related to the fact that newborns from triplet pregnancies have high rates of preterm labor and premature rupture of membranes, even though in many cases, preterm termination of pregnancy is scheduled [59]. In either case, it is not surprising that corticosteroids for antenatal lung maturity are frequently administered. Prematurity is also related to low birth weight (more than a third showed low birth weight and 11.47% presented very low birth weight) and with lower average APGAR scores, although most recover at minute 5 as a result of the advanced neonatal and resuscitation care and resources available today.

In our meta-analysis, regarding the mode of delivery, there were no significant differences based on chorionicity, as expected, given that the current guidelines recommend delivery by scheduled cesarean section regardless of chorionicity [30]. Nevertheless, a trend towards greater prematurity and lower birth weight was observed in mono- or dichorionic triplet pregnancies compared to trichorionic pregnancies. These findings align with what has been known to occur in twin pregnancies, although our analysis did not reach statistical significance.

#### 4.2.4. Neonatal Outcomes

In the systematic review, 78% of newborns were admitted to the NICU, the majority due to respiratory morbidity. Surfactant was administered to one-fifth (22.7%) of the newborns, and one-third (34.49%) required mechanical ventilation. As mentioned previously, these findings are consistent with the significant prematurity of newborns from triplet pregnancies.

In our meta-analysis, admission to the NICU in newborns of TCTA pregnancies was almost 50% compared to those of non-TCTA pregnancies (OR 0.57; 95% CI 0.44–0.72), which could be related to the trend, which did not achieve statistical significance, of lower risk for prematurity and low birth weight, hence with the lower risk of other neonatal complications. It was observed that newborns of TCTA pregnancies have less than half the risk of developing respiratory distress, which is the main complication of these newborns [37,41,48]. However, neither the administration of surfactant nor bronchopulmonary dysplasia were influenced by the chorionicity of triplet pregnancy, possibly due to their low prevalence and the few articles that reported these variables.

Other neonatal complications resulting from the increased prematurity in triplet pregnancies, are sepsis (5.48%), severe intraventricular hemorrhage (4.6%), retinopathy of the premature (4.12%) and necrotizing enterocolitis (1.67%). [14,24,28,29]. The risk of developing sepsis and necrotizing enterocolitis was higher in newborns from non-TCTA triplet pregnancies, while there were no differences for severe intraventricular hemorrhage or retinopathy. These last two events are rare events, and it is possible that greater statistical power is required to reach statistical significance. Regarding retinopathy, perhaps the inclusion of the article by Kawaguchi et al. [14] that found no significant differences in this disease despite a large study sample prevented the global trend from being statistically significant.

#### 4.2.5. Mortality

Regarding the results of the systematic review, it is striking that miscarriages before 22 weeks were only reported for 2.88% of the fetuses included, and in fact, 32 studies excluded them from their analysis, so the interpretation of this data should be cautious. Intrauterine mortality was also excluded from 16 articles, although it was collected in 26.17% of the pregnancies included, and neonatal mortality was only collected in 34.41% of the live newborns included in the review. Even so, the sample on which these variables were reported was between 40,000 and 44,000 fetuses, so we consider that the data obtained can be considered representative. Intrauterine fetal mortality was found to affect 5% of all fetuses in triplet pregnancies, and neonatal mortality in 4.42% of newborns.

According to the results of our meta-analysis, intrauterine mortality is decreased in fetuses from TCTA pregnancies compared to those from non-TCTA pregnancies [14,16,18,21,24,33,41]. The pathophysiology of a shared placenta may be the underlying explanation of our findings, given the risk of developing TTTS, restricted intrauterine growth, anemia-polycythemia sequence, and cardiovascular compromise in monoamniotic fetuses [14,16,80].

As for neonatal mortality, we found no statistically significant differences when comparing TCTA pregnancies with non-TCTA pregnancies. One possible explanation for this finding would be that, despite greater neonatal morbidity in terms of the previously described complications, access to NICUs and high-complexity centers makes it possible to reduce neonatal mortality without differentiating newborns based on their chorionicity. However, there seems to be a certain temporal trend in the results of neonatal mortality in the articles that report it, showing higher neonatal mortality in older studies. This could be explained by the progress made in fetal surgery and interventionism in the last decade.

The risk of perinatal mortality was also shown to be significantly increased in fetuses and newborns from non-TCTA pregnancies, probably at the expense of intrauterine mortality.

Finally, there is a notable lack of data on maternal mortality in triplet pregnancies. Only four studies reported maternal mortality as a study outcome, which was an isolated case of maternal death out of a total of 721 pregnant women included in these studies (0.14%) [35,39,53,55]. Similarly, only one study reported a composite variable on maternal morbidity [34], which was present in 32 of the 35 patients on which this data was reported. It is surprising how little thought is given to maternal mortality and morbidity in studies on triplet pregnancies, especially when increased maternal mortality has been described in multiple pregnancies [81]. In fact, multiple pregnancies are risk factors for serious maternal complications, such as hypertensive disorders of pregnancy [17] or postpartum hemorrhages [82]. Maternal mortality, although less and less frequent in our environment, is always a tragic event, and in the case of multiple pregnancies, we cannot forget the scope of its repercussions. The loss of a mother in a triplet pregnancy results in three newborns losing a parent, with the social, economic, and emotional consequences that this might entail, thus tripling its impact [83].

### 4.3. Strengths

To our knowledge, this is the largest systematic review and meta-analysis conducted to date on maternal, fetal, and neonatal morbidity and mortality of triplet pregnancies and their relationship to chorionicity. One of the main strengths of this study is the gathering of such a large population sample from an event as rare as triplet pregnancies.

As mentioned above, the main difference with respect to the meta-analysis published by Curado et al. [18] is that they focused on variables related to fetal and neonatal mortality, including fewer composite secondary variables, and fewer studies (and consequently, smaller population sample and data) for analysis. Our meta-analysis, on the one hand, provides robust and comprehensive evidence to the common results with which it aligns, since it includes more data from more studies. On the other hand, this study adds richness and diversity to the currently published data since it does not focus exclusively on neonatal or mortality outcomes but also analyzes maternal, gestational, and obstetric outcomes.

Finally, the authors believe that conducting a systematic review prior to performing the meta-analysis is also one of the strengths of this article. It provides the clinician with a highly applicable global vision for the risks and complications that may occur in these high-risk pregnancies. It also facilitates the interpretation of the results of the meta-analysis on the impact that chorionicity has on morbidity and mortality. As it has been suggested, the subgroup analysis according to chorionicity is of special relevance, both for patient counsel as well as for obstetric clinical management.

### 4.4. Limitations

The main limitations of our meta-analysis are related to the retrospective observational designs of the studies included, their relatively small population samples, and their high heterogeneity. Finally, the publication bias analyzed using Egger’s test in the variables included in the meta-analysis could be present in at least four of them.

Additionally, the review includes articles from 2005 to 2020, an extended period of time during which the management of triplet pregnancies and their newborns may have shifted substantially.

Another limitation is the lack of information not reported by numerous studies about variables that the authors considered relevant when evaluating morbidity and mortality in triplet pregnancies. Few articles describe complications intrinsic to multiple pregnancies, such as TTTS or postpartum hemorrhage. The current meta-analysis has evaluated the influence of chorionicity on fetal, maternal, and perinatal outcomes. However, other factors such as the mode of conception (different ARTs and spontaneous conception) have not been studied in detail in our meta-analysis. Addressing how fetal, perinatal, and maternal morbidity and mortality in triplet pregnancies might differ when comparing the different modes of conception is an interesting question for further research.

Finally, we consider that maternal mortality is a relevant outcome that should not be left in the background when addressing the morbidity and mortality of triplet pregnancies. Indeed, triplet pregnancies have been found to be, not only high-risk events for the newborns but also for the mothers themselves.

Finally, the authors are aware that recent studies that might have been published after finishing our systematic review, such as Badreldin et al. [84] and Mitsiakos et al. [85], were not included in this article and should be taken into account in future research.

## 5. Conclusions

The results of the systematic review described a high incidence of maternal, fetal, and obstetric morbidity, largely related to the prematurity of triplet pregnancies. In our meta-analysis, we observed a significant increase in maternal, fetal, and neonatal morbidity and mortality in non-TCTA triplet pregnancies compared to TCTA. Further research is needed to assess some of the outcomes that either could not be included in our study due to the scarcity of publications reporting them, or in which statistical significance was not reached, probably due to an insufficient sample size.

## Figures and Tables

**Figure 2:**
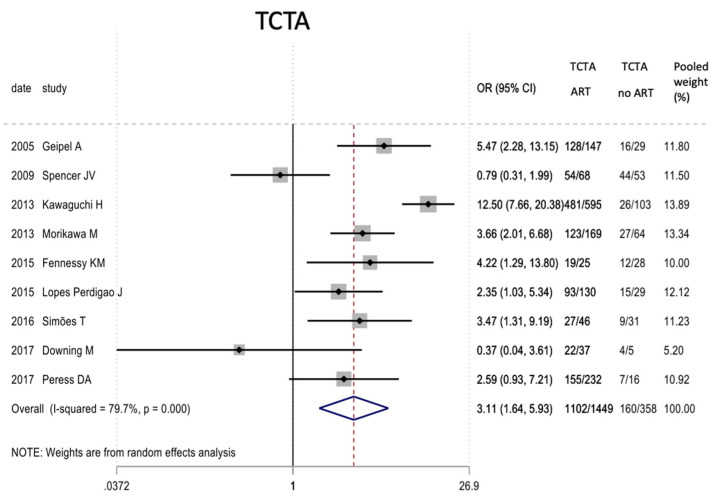
Results of the meta-analysis on the effect of assisted reproductive therapies on the chorionicity of pregnancies (TCTA vs. non-TCTA). TCTA: trichorial-triamniotic, non-TCTA: non-trichorial-triamniotic. OR = odds ratio, CI = confidence interval.

**Figure 3:**
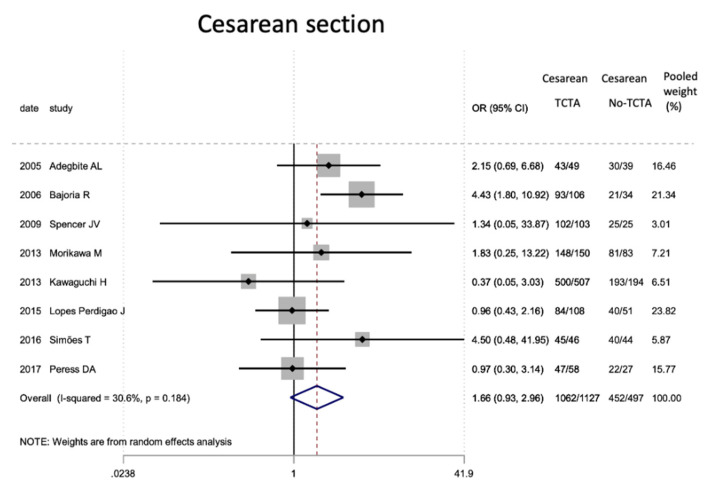
Results of the meta-analysis on the effect of chorionicity (TCTA vs no TCTA) on cesarean as the mode of delivery. TCTA: trichorial-triamniotic, non-TCTA: non-trichorial-triamniotic. OR = odds ratio, CI = confidence interval.

**Figure 4:**
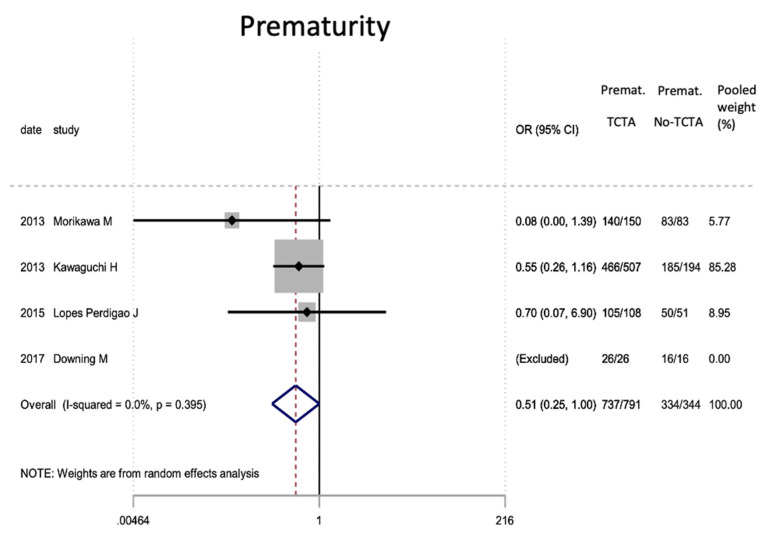
Results of the meta-analysis on the effect of chorionicity (TCTA vs no TCTA) on prematurity in newborns (under 37 weeks). Premat: prematurity, TCTA: tricorial-triamniotic, non-TCTA: non-tricorial-triamniotic. OR = odds ratio, CI = confidence interval.

**Figure 5:**
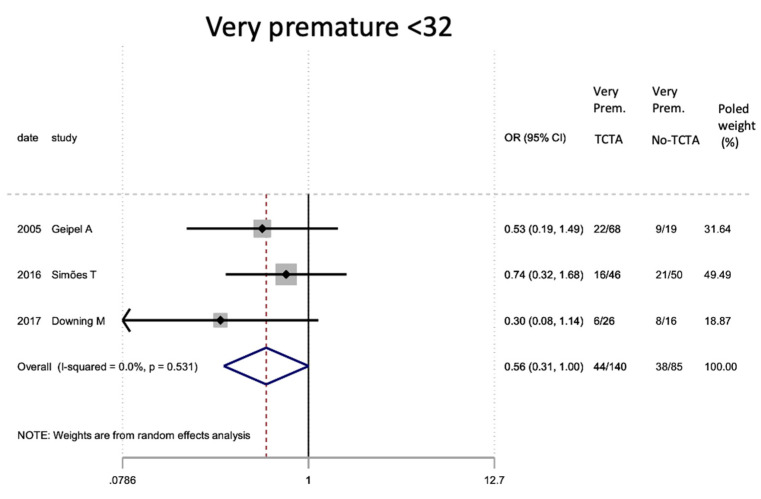
Results of the meta-analysis on the effect of chorionicity (TCTA vs no TCTA) on very preterm newborns (under 32 weeks). Very Prem: very premature; TCTA: trichorial-triamniotic, non-TCTA: non-trichorial-triamniotic. OR = odds ratio, CI = confidence interval.

**Figure 6:**
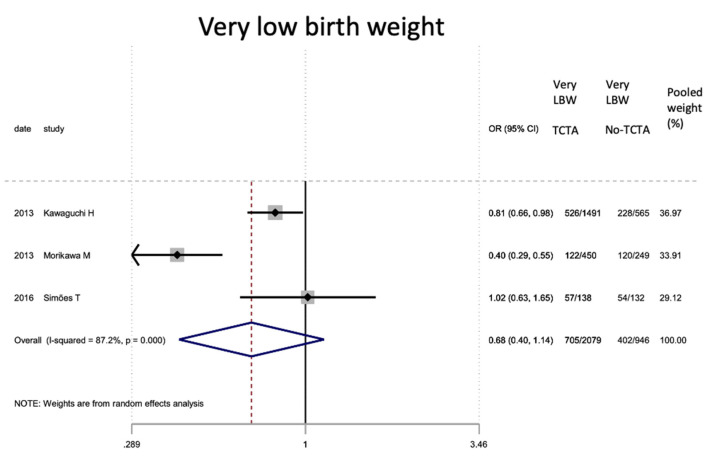
Results of the meta-analysis on the effect of chorionicity (TCTA vs no TCTA) on very low birth weight newborns (below 1500 g). Very LBW: very low birth weight; TCTA: trichorial-triamniotic, non-TCTA: non-trichorial-triamniotic. OR = odds ratio, CI = confidence interval.

**Figure 7:**
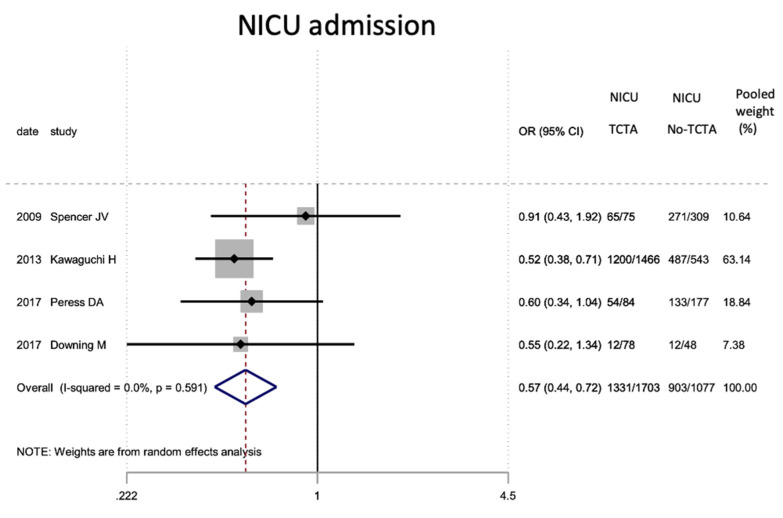
Results of the meta-analysis on the effect of chorionicity (TCTA vs no TCTA) on NICU admission. NICU: neonatal inten-sive care unit; TCTA: trichorial-triamniotic, non-TCTA: non-trichorial-triamniotic. OR = odds ratio, CI = confidence interval.

**Figure 8:**
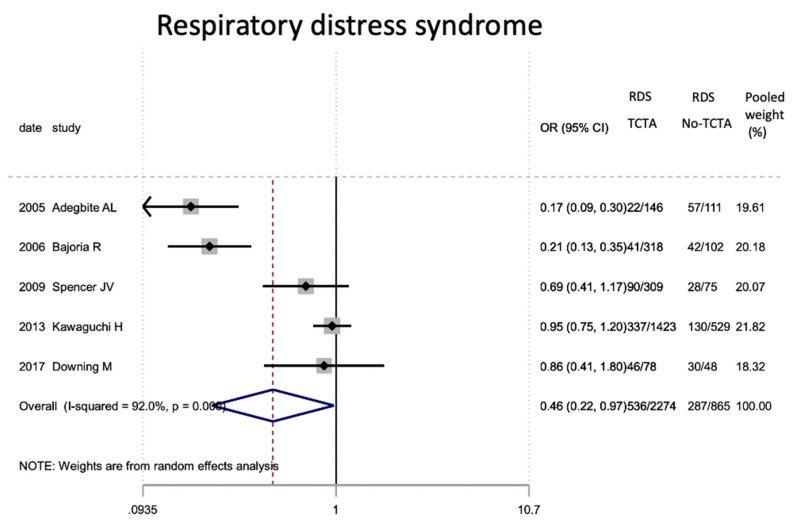
Results of the meta-analysis on the effect of chorionicity (TCTA vs no TCTA) on neonatal respiratory distress. RDS: respiratory distress; TCTA: trichorial-triamniotic, non-TCTA: non-trichorial-triamniotic. OR = odds ratio, CI = confidence interval.

**Figure 9:**
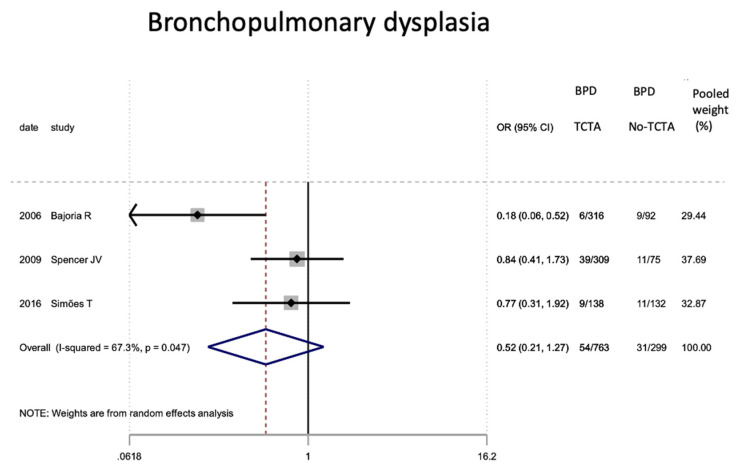
Results of the meta-analysis on the effect of chorionicity (TCTA vs no TCTA) on the development of neonatal bronchopulmonary dysplasia. BPD: bronchopulmonary dysplasia; TCTA: trichorial-triamniotic, non-TCTA: non-trichorial-triamniotic. OR = odds ratio, CI = confidence interval.

**Figure 10:**
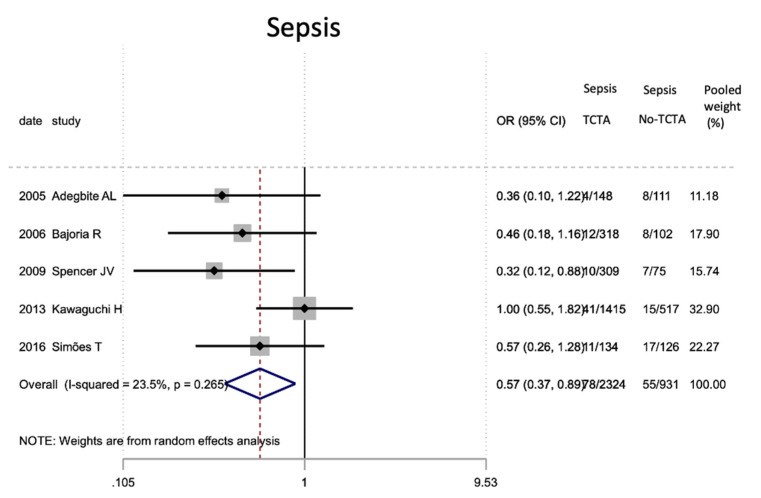
Results of the meta-analysis on the effect of chorionicity (TCTA vs no TCTA) on the development of neonatal sepsis; TCTA: trichorial-triamniotic, non-TCTA: non-trichorial-triamniotic. OR = odds ratio, CI = confidence interval.

**Figure 11:**
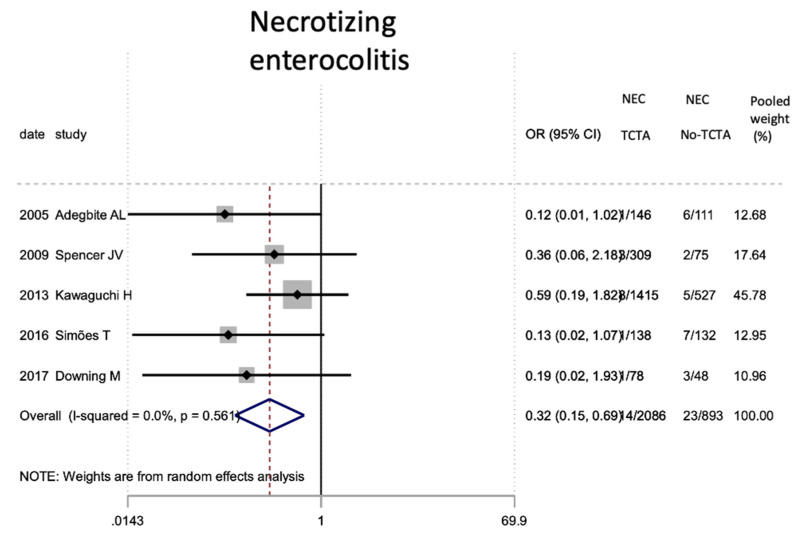
Results of the meta-analysis on the effect of chorionicity (TCTA vs no TCTA) on the development of neonatal necrotizing enterocolitis. NEC: necrotizing enterocolitis; TCTA: trichorial-triamniotic, non-TCTA: non-trichorial-triamniotic. OR = odds ratio, CI = confidence interval.

**Figure 12:**
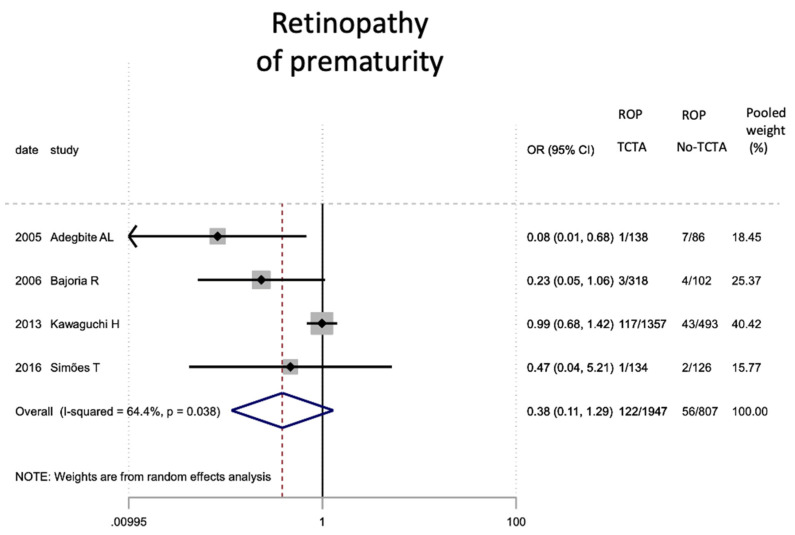
Results of the meta-analysis on the effect of chorionicity (TCTA vs no TCTA) on the development of retinopathy of prem-aturity. ROP: retinopathy of prematurity; TCTA: trichorial-triamniotic, non-TCTA: non-trichorial-triamniotic. OR = odds ratio, CI = confidence interval.

**Figure 13:**
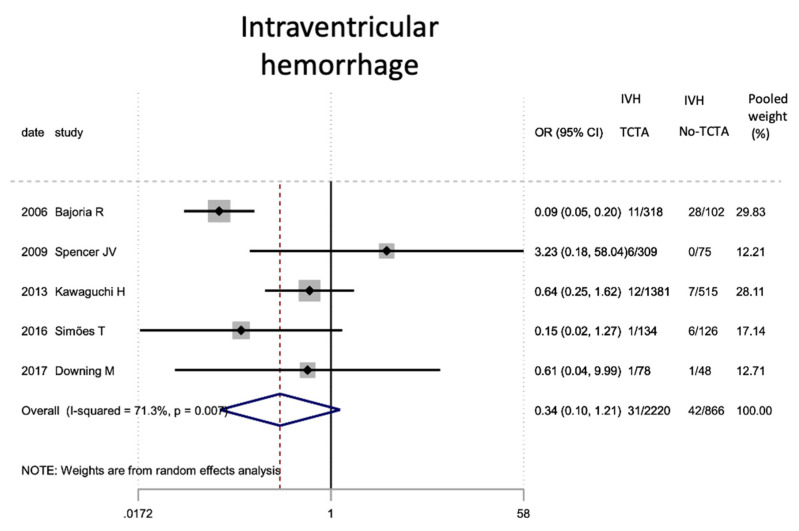
Results of the meta-analysis on the effect of chorionicity (TCTA vs no TCTA) on the development of severe stage III-IV intraventricular hemorrhage. IVH: intraventricular hemorrhage; TCTA: trichorial-triamniotic, non-TCTA: non-trichorial-triamniotic. OR = odds ratio, CI = confidence interval.

**Figure 14:**
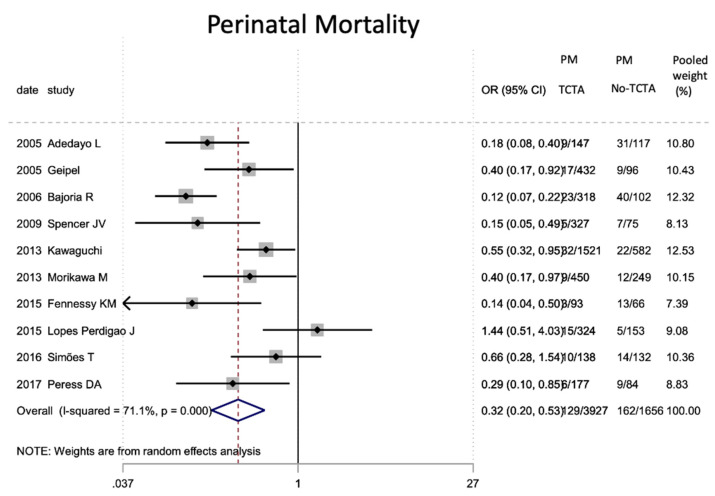
Results of the meta-analysis on the effect of chorionicity (TCTA vs no TCTA) on perinatal mortality. PM: perinatal mortal-ity; TCTA: trichorial-triamniotic, non-TCTA: non-trichorial-triamniotic. OR = odds ratio, CI = confidence interval.

**Figure 15:**
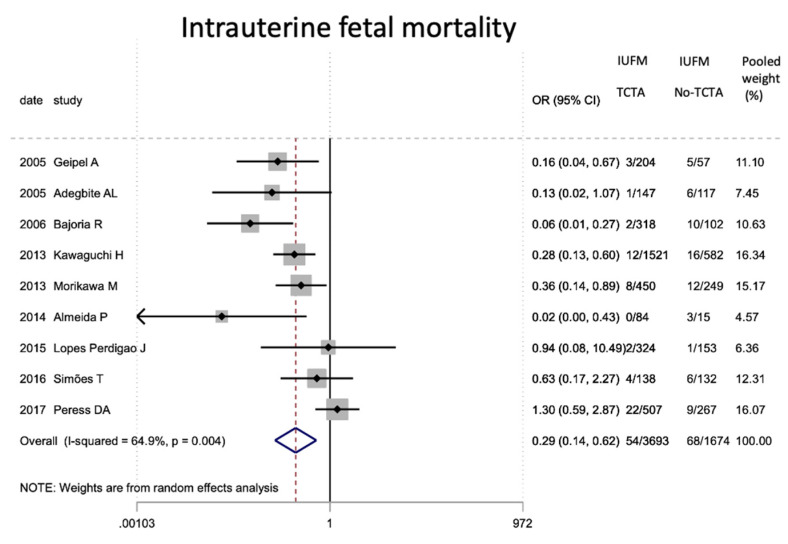
Results of the meta-analysis on the effect of chorionicity (TCTA vs no TCTA) on intrauterine fetal mortality. MFIU: intrau-terine fetal mortality; TCTA: trichorial-triamniotic, non-TCTA: non-trichorial-triamniotic. OR = odds ratio, CI = confidence interval.

**Figure 16:**
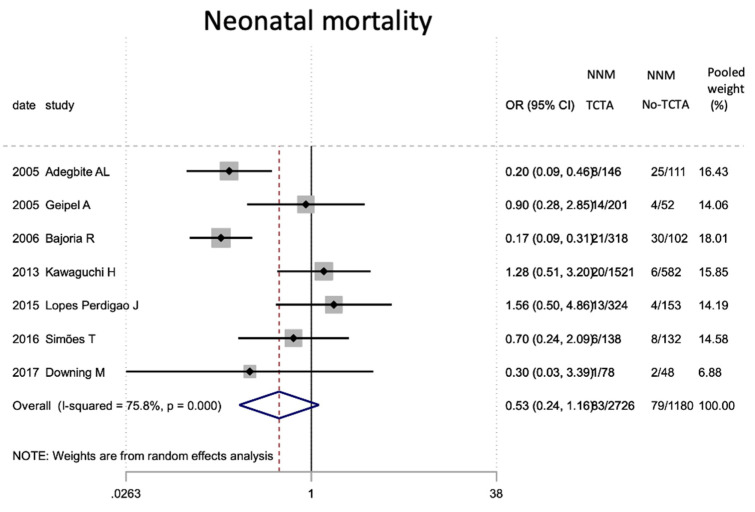
Results of the meta-analysis on the effect of chorionicity (TCTA vs no TCTA) on neonatal mortality. NNM: neonatal mor-tality; TCTA: trichorial-triamniotic, non-TCTA: non-trichorial-triamniotic. OR = odds ratio, CI = confidence interval.

**Table 1:** Characteristics of the studies included in the systematic review and meta-analysis.

Number	Authors	Country	Year of Publication	Recruitment Period	Study Type	Center	Live Newborn	Triplet Pregnancies
1	Adegbite et al. *	United Kingdom	2005	1986–2000	ROC	M	257	88
2	Day et al.	USA	2005	1995–2004	POC	M	7024	2545
3	Geipel et al. *	Germany	2005	1998–2003	ROC	M	253	176
4	Salihu, Aliyu et al.	USA	2005	1995–1997	PS	M	15,021	5265
5	Salihu, Bagchi et al.	USA	2005	1995–1998	PS	M	21,676	7225
6	Al-Suleiman et al.	Saudi Arabia	2006	1990–2004	ROC	U	104	35
7	Bajoria et al. *	United Kingdom	2006	1986–2000	ROC	M	408	140
8	Luke et al.	USA	2006	1996–2001	ROC	U	24	8
9	Eddib et al.	USA	2007	1999–2003	ROC	U	NR	56
10	Luke et al.	USA	2007	1989–2001	PS	M	26,829	8943
11	Luke et al.	USA	2007	1995–2000	PS	M	36,579	12,193
12	Zuppa et al.	Italia	2007	1994–2003	ROC	U	71	24
13	Mazhar et al.	Pakistan	2008	2000–2006	ROC	U	50	18
14	Battin et al.	New Zealand	2009	1995–2005	ROC	U	155	55
15	Kraemer et al.	Germany	2009	1980–1997	ROC	U	77	26
16	Spencer et al. *	USA	2009	1995–2007	ROC	M	379	128
17	Tandberg et al.	Norway	2010	1967–2006	ROC	M	1344	448
18	Al-Sunaidi et al.	Saudi Arabia	2011	2007–2009	ROC	U	87	32
19	Arlettaz et al.	Switzerland	2011	2005–2008	ROC	M	290	100
20	Machtinger et al.	Israel	2011	1997–2005	ROC	U	219	73
21	Moore et al.	USA	2012	1989–2010	POC	U	417	139
22	Chibber et al.	Kuwait	2013	2001–2011	ROC	U	278	100
23	Kawaguchi et al. *	Japan	2013	1999–2009	ROC	M	2076	701
24	Morikawa et al. *	Japan	2013	2005–2008	ROC	M	960	320
25	Revello et al.	Spain	2013	2000–2010	ROC	U	428	147
26	Weissman et al.	Israel	2013	2001–2011	ROC	U	102	34
27	Almeida et al. *	Portugal	2014	1996–2011	POC	U	96	33
28	Fennessy et al. *	Australia	2015	1999–2011	ROC	U	150	53
29	Lopes et al. *	USA	2015	1999–2010	ROC	M	474	159
30	Lappen et al.	USA	2016	2002–2008	ROC	M	240	80
31	Maia et al.	Brazil	2016	1998–2012	ROC	U	185	67
32	Morency et al.	Canada	2016	2000–2013	ROC	U	690	230
33	Simões et al. *	Portugal	2016	1994–2014	ROC	U	260	90
34	AlBasri et al.	Saudi Arabia	2017	2004–2011	ROC	U	62	21
35	Downing et al. *	USA	2017	2009–2015	ROC	U	123	42
36	Lachowska et al.	Polonia	2017	2006–2015	ROC	U	99	34
37	Peress et al. *	USA	2017	2005–2016	ROC	U	NR	258
38	Razavi et al.	USA	2017	2003–2015	ROC	U	240	80
39	Ko et al.	South Korea	2018	2009–2015	PS	M	1865	621
40	Rajan et al.	India	2018	2000–2014	ROC	U	225	82
41	Shah et al.	Intercontinental	2018	2007–2013	ROC	M	6079	2026
42	Shah et al.	USA	2018	2004–2006	ROC	M	381	127
43	Dudenhausen et al.	Europe	2019	2011–2012	PS	M	258	97
44	Kyeong et al.	South Korea	2019	1992–2012	ROC	M	185	65
45	Mol et al.	Netherlands	2019	1999–2008	ROC	M	1158	386
46	Peress et al.	USA	2019	2005–2016	ROC	U	249	83
	All articles included in sistematic review						128,127	43,653
	All articles included in the meta-analysis *						5441	2188

ROC: retrospective observational cohort study; POC: prospective observational cohort study. PS: population study. M: multicentric; U: unicentric; NR: non reported. (*) All articles included in the meta-analysis.

**Table 2:** Maternal and gestational characteristics of the triplet pregnancies included in the systematic review.

Maternal and Gestational Characteristics	Number of Studies	Number of Pregnancies	*n* (%) or Mean
Maternal age (median, SD)	39	28,415	31.6
Primipara	31	32,537	14,890 (45.76%)
BMI	12	1858	26.7
Maternal medical conditions	7	18,194	832.4 (4.58%)
Assisted reproductive technology	31	4352	2962 (68.06%)
Triamniotic-trichorionic	25	3616	2318 (64.10%)
Triamniotic-dichorionic	22	3276	815 (24.88%)
Triamniotic-monochorionic	21	3035	129 (4.25%)

BMI: body mass index.

**Table 3:** Obstetric complications in the triplet pregnancies included in the systematic review.

Obstetric Complications	Number of Studies	Number of Pregnancies	*n* (%)
Antenatal corticosteroids	19	4310	3336 (77.4%)
Cervical cerclage	11	1366	566 (41.43%)
Intrauterine growth restriction/low birth weight	19	13,989	4884.8 (34.92%)
Threatened preterm labor	24	14,848	3918.7 (26.39%)
Hipertensive disorders	34	25,571	3626.7 (14.18%)
Preterm rupture of membranes	25	14,997	1725.8 (11.51%)
Diabetes	22	22,764	1493.6 (6.56%)
Fetal malformation	17	1866	122 (6.54%)
Postpartum hemorrhage	7	12,764	584.6 (4.58%)
Twin to twin transfusion syndrome	18	2649	74 (2.79%)
Antenatal bleeding	10	12,914	315.8 (2.45%)

**Table 4:** Perinatal results of the triplet pregnancies included in the systematic review.

Perinatal Results	Number of Studies	Number of Pregnancies * or Newborns ^+^	*n*(%) or Mean
Cesarean section	34	23,791 *	21,169.8 (88.98%)
GA at delivery	41	22,247 *	32.3
GA <37weeks	16	24,001 *	22,284.7 (92.85%)
GA <34 weeks	8	1537 *	885 (57.58%)
GA <32 weeks	17	11,136 *	4559.6 (40.94%)
GA <28 weeks	15	11,418 *	1475.7 (12.92%)
Birth weight (g)	41	28,529 ^+^	1638
Very low birth weight (<1500 g)	11	41,383 ^+^	14,375 (34.74%)
Extremely low birth weight (<1000 g)	7	4403 ^+^	505 (11.47%)
APGAR 1min	7	1113 ^+^	6.8
APGAR 5min	9	1526 ^+^	8.1
APGAR 5min <7	10	24,781 ^+^	2076 (8.38%)
Male	8	8573	4194 (48.92%)

GA: gestational age. (*): Total number of pregnancies for which this outcome measure has been reported. (**^+^**): Total number of newborns for which this outcome measure has been reported.

**Table 5:** Neonatal results of the triplet pregnancies included in the systematic review.

Neonatal Results	Number of Studies	Number of Newborns	*n* (%)
Neonatal intensive care unit	17	5308	4177 (78.69%)
Respiratory distress	21	6685	1906 (28.51%)
Hyaline membrane disease	3	191	84 (43.98%)
Surfactant administration	5	3212	729 (22.7%)
Bronchopulmonary dysplasia	15	10,148	997 (9.82%)
Assisted ventilation	16	5845	2016 (34.49%)
Sepsis	15	5652	310 (5.48%)
Necrotizing enterocolitis	17	6032	102 (1.69%)
Retinopaty	14	10,708	440.9 (4.12%)
Intraventricular hemorrhage III-IV	22	12,923	601 (4.65%)
Mortality composite	12	9336	2103 (22.53%)

**Table 6:** Mortality and morbidity results of the triplet pregnancies included in the systematic review.

Mortality and Morbidity	Number of Studies	Number of Pregnancies *, Fetus or Newborns ^+^	*n* (%)
Miscarriage <22 weeks	9	3870 *	187 (4.83%)
Intrauterine mortality	29	40,347 ^+^	2015 (5%)
Neonatal mortality	34	44,089 ^+^	1947.4 (4.42%)
Perinatal mortality	31	40,575 ^+^	700 (1.73%)
Maternal mortality	6	721 *	1 (0.4%)
Maternal morbidity	1	35 *	32 (91.43%)

(*): Total number of pregnancies for which this outcome measure has been reported. (+): Total number of newborns for which this outcome measure has been reported.

**Table 7:** Maternal y perinatal characteristics of the triplet pregnancies included in the meta-analysis according to chorionicity.

Maternal and Perinatal Characteristics	Number of Studies	Number of Pregnancies * or Newborns ^+^	Combined OR (CI 95%)	OR Test (*p*)	Heterogenenicity 12%	Heterogeneicidad χ^2^ (*p*)	Egger Test (*p*)
Reproductive assited techniques	9	1807 *	3.115 (1.635–5.933)	0.001 ^#^	79.7	0.000	0.034 ^#^
Cesarean section	8	1624 *	1.658 (0.928–2.965)	0.088	30.6	0.184	0.846
GA <37 weeks	3	1135*	0.51 (0.25–1.00)	0.051	0.0	0.395	0.581
GA <32 weeks	3	225*	0.56 (0.31–1.00)	0.051	0.0	0.531	0.032 ^#^
Very low birth weight (<1500 g)	3	3025^+^	0.68 (0.40–1.14)	0.144	87.2	0.000	0.892

^#^ statistical significance *p* < 0.05. OR = odds ratio. (*): Total number of pregnancies for which this outcome measure has been reported. (**^+^**): Total number of newborns for which this outcome measure has been reported.

**Table 8:** Neonatal results of the triplet pregnancies included in the meta-analysis according to chorionicity.

Neonatal Results	Number of Studies	Number of Newborns	Combined OR (CI 95%)	OR Test (*p*)	Heterogenenicity 12%	Heterogeneicidad χ^2^ (*p*)	Egger Test (*p*)
Neonatal intensive care unit	4	2780	0.57 (0.44–0.72)	0 ^#^	0.0	0.591	0.332
Respiratory distress	5	3139	0.46 (0.22–0.97)	0.043 ^#^	92	0.000	0.239
Hyaline membrane disease	3	1062	0.52 (0.21–1.27)	0.151	67.3	0.047	0.371
Sepsis	5	3255	0.57 (0.37–0.89)	0.013 ^#^	23.5	0.265	0.016 ^#^
Necrotizing enterocolitis	5	2979	0.32 (0.15–0.69)	0.003 ^#^	0.0	0.561	0.022 ^#^
Retinopaty	4	2754	0.38 (0.11–1.29)	0.123	64.4	0.038	0.108
Intraventricular hemorrhage III–IV	5	3086	0.34 (0.10–1.21)	0.097	71.3	0.007	0.386

^#^ statistical significance *p* < 0.05. OR = odds ratio.

**Table 9:** Mortality results of the triplet pregnancies included in the meta-analysis according to chorionicity.

Mortality	Number of Studies	Number of Live Newborns	Combined OR (CI 95%)	OR Test (*p*)	Heterogenenicity 12%	Heterogeneicidad χ^2^ (*p*)	Egger Test (*p*)
Intrauterine mortality	9	5367	0.29 (0.14–0.62)	0.001 ^#^	64.9	0.004	0.165
Neonatal mortality	7	3906	0.53 (0.24–1.16)	0.114	75.8	0.000	0.224
Perinatal mortality	10	5583	0.32 (0.20–0.53)	<0.001 ^#^	71.1	0.000	0.882

^#^ statistical significance *p* < 0.05. OR = odds ratio.

## Data Availability

Not applicable.
